# The association of VDR polymorphisms and type 2 diabetes in older people living in community in Santiago de Chile

**DOI:** 10.1038/s41387-018-0038-9

**Published:** 2018-05-25

**Authors:** Bárbara Angel, Lydia Lera, Carlos Márquez, Cecilia Albala

**Affiliations:** Public Nutrition Unit, Institute of Nutrition and Food Technology (INTA), University of Chile, Santiago, Chile

## Abstract

**Introduction:**

Several polymorphisms have been associated with obesity and type 2 diabetes in different populations.

**Objective:**

To investigate the frequencies of a genetic polymorphism of vitamin D receptor (FokI and BsmI) in patients with T2D.

**Methods:**

The case–control study was conducted in 138 patients with T2D and 172 control subjects, men and women (60–79 years old). The genotype and allele frequency determination of VDR polymorphisms were determined in these subjects.

**Results:**

The frequency of the C allele of the FokI polymorphism was significantly higher in the T2D group than in healthy subjects (*p* = 0.025). The frequencies of the BsmI variant were similar in subjects with and without T2D (*p* = 0.747). Consistent with these data, there was an association of the C allele with T2D (OR = 1.74, 95% CI = 1.003–3.084, *p* = 0.036), but not the AG + GG variants for BsmI (OR = 1.02, 95% CI = 0.635–1.649, *p* = 0.916). We can observe a significant association between carrier of the T > C variant of FokI and type 2 diabetes, adjusted for vitamin D, age, obesity (overweight and obesity), seasonality, sex and Homa-IR. Here, we show a significant association between the FokI polymorphisms (TC + CC) and T2D with an odds ratio of 1.9001 (95% CI (1.0970–3.6838), *p* = 0.041).

**Conclusion:**

Our study suggests that the C allele (TC + CC) of the VDR-FokI gene is a possible risk factor for T2D in older people living in a community in Santiago de Chile.

## Introduction

The role of micronutrients is fundamental in the metabolic processes of living beings. In the case of vitamin D, it plays a central role in the metabolism of bone and calcium homeostasis, and also exerts an effect on the growth and differentiation of diverse cell types. On the other hand, it shows immunoregulatory properties, meaning that it participates in the development of several chronic diseases^1,2^. Vitamin D deficiency is common, especially in older adults in whom the ability to synthesize vitamin D is diminished^3^.

Different studies have reported an inverse association between plasma levels of vitamin D (vitD) and the risk of diabetes, insulin resistance and metabolic syndrome^4–7^. Clinical studies focused on type 2 diabetes (T2D), have shown a positive correlation between circulating vitD levels and insulin sensitivity, indicating that vitD deficiency may predispose to glucose intolerance, the secretion of altered insulin and T2D^8–10^, either through a direct action via activation of the vitamin D receptor (VDR), or indirectly through calcemic hormones and also through inflammatory processes^1,11,12^.

Low-intensity chronic inflammation, often seen in obese individuals, is involved in the development of insulin resistance, which increases the risk of T2D^13^. There is strong evidence that the activation of inflammatory pathways interferes with the normal metabolism of insulin and alters its signaling, resulting in an increased expression of pro-inflammatory cytokines^14^. These cytokines can be directed to cell membrane receptors, feeding the inflammatory response and exacerbating insulin resistance^15^. Epidemiological studies have shown that tumor necrosis factor-α, C-reactive protein (CRP) and interleukin-6 are positively correlated with body mass index (BMI) and body fat percentage^6,16^. Recent studies have associated vitD status with the development of T2D^16,17^, although the mechanisms underlying the role of vitD in T2D are still partially explained.

The VDR is widely distributed^18^, controlling genes related to bone metabolism, oxidative damage, chronic diseases and inflammation^19^. VitD and its receptor complex play a role in the regulation of insulin secretion from beta cells^20,21^. However, the genetic component of T2D remains unclear; it has been shown that some of these polymorphisms are associated with T2D, insulin secretion^22,23^, as well as with the metabolic changes related to obesity^24^. Several polymorphisms, such as BsmI and FokI, have been described in VDR genes that are capable of altering the activity of the VDR protein^24,25^. The most frequent VDR Single nucleotide polymorphisms (SNPs) are FokI, BsmI, TaqI and ApaI. Of these SNPs, the BsmI and ApaI polymorphisms were the result of substitutions in intron 8, whereas Taq1 resulted from a substitution of cytosine (C) by thymine (T) in exon 9. These three SNPs (BsmI, ApaI and TaqI) are close to 3 ‘-3’ -Region Region not translated (3’-UTR) and is believed to alter the stability of the VDR mRNA. The FokI polymorphism corresponds to a polymorphic site in exon 2. More specifically, a variation of T to C at the start codon of the VDR gene The change from ATG (FokI T) to ACG (FokI C) results in a VDR protein that is three amino acids shorter compared with the full-length VDR, this shorter form may have altered biological activity^25,26^.

Human studies investigating the influence of genetic vitD state of VDR SNPs on inflammatory biomarkers in subjects with T2D and its complications are rare and have generated conflicting results. This work aims to investigate the association of VDR polymorphisms [VDR rs2228570 (FokI) and rs1544410 (BsmI)] with T2D and its metabolic components in older adults living in a community in Santiago de Chile.

## Methodology

### Subjects

The study was conducted between 2010 and 2011 using a case–control type design. The sample included subjects aged from 60 to 79 years old, of medium and low socioeconomic backgrounds, living in the community. Both case and controls groups were randomly selected from registries of primary care centers of the city of Santiago de Chile. The case group was composed of 138 people with T2D, and the control group included 172 comparable subjects, without diabetes.

During the study period, the 310 subjects were invited to attend an appointment at the Institute of Nutrition and Food Technology of the University of Chile (INTA) for an evaluation; all individuals were interviewed by a specially trained professional, and a questionnaire containing questions about sociodemographic background and history of chronic diseases was applied. An anthropometric evaluation was then carried out (height, weight and waist circumference) and a blood sample was obtained. For the diagnosis of T2D, the criteria of the American Diabetes Association were used. Exclusion criteria were autoimmune diseases, chronic renal failure and self-reported vitD supplement consumption in the last 6 months. The INTA Ethics Committee, University of Chile, approved the study protocol that it conforms to the provisions of the Declaration of Helsinki. All participants signed an informed consent after receiving written and verbal information about the study.

### Anthropometric measurements

The anthropometric measurements of height, weight and waist circumference were taken by specially trained personnel for the study, with procedures previously described by our group^3,27^.

### Biochemical measurements

Blood samples (10 ml total) were taken in the morning between 07:00 and 09:00 after an overnight fast of 12 h. The samples were centrifuged and kept stored at −80 °C until analysis. Serum levels of glucose, insulin, total cholesterol and high-density lipoprotein (HDL) cholesterol, triglycerides, ultra-sensitive C-reactive protein (US-CRP) and 25-hydroxyvitamin D3 (25(OH)D3) were measured. Blood samples were collected throughout the year, except for February (University break).

Plasma levels of 25(OH)D3 were determined by radioimmunoassay (DiaSorin Stillwater, Minnesota 55082-0285, USA) with quality control materials provided by the manufacturer. The limit of detection for 25(OH)D3 was 6 nmol/l, the intra-assay coefficient of variation (CV) was 10.8% and the inter-assay CV was 9.4%. Insulin was determined by RIA, DPC, INC, The Los Angeles, CA. KIT Coat-a-Count, the intra-assay CV was 5.2% and the inter-assay CV was 7.3. The kit determined plasma levels of US-CRP by the Immunoturbidimetry Method: Latex High sensitivity CRP Turbidimetric (Química Clínica Aplicada SA, QCA, CN-Section 20-E43870 Amposta/Spain). Serum glucose, triglycerides, total cholesterol and HDL cholesterol were measured by enzymatic colorimetric assay. All coefficients of intra- and inter-assay variation were <10%.

Diabetes was defined as fasting glycemia values ≥126 mg/dl or the use of anti-diabetic agents; these subjects with T2D are not insulin dependent. The vitD status was defined according to the proposal by the Endocrine Society in 2011, where they suggest a minimum concentration of 75 nmol/L (30 ng/ml)^28^, a deficiency to be indicated by serum 25(OH)D3 < 50 nmol/L (<20 ng/ml) and vitD insufficiency to be denoted by 50–74 nmol/L (20–30 ng/ml)^29,30^. Obesity was defined according to the WHO criteria (BMI ≥ 30 kg/m^2^) and insulin resistance as Homeostatic model assessment of insulin resistance (HOMA-IR) HOMA-IR ≥ 2.6^31^.

### Genetic analysis of polymorphisms in VDR

Genomic DNA was extracted from leukocytes by standardized methods (GIBCO BRL, USA). Genotypes for the polymorphic restriction sites of the VDR gene rs2228570 (FokI) and rs1544410 (BsmI) were obtained by DNA amplifications with standard PCR and specific sets of starters, followed by the RFLP method according to previous protocols used by our group^32–34^.

### Statistical methods

All statistical analyses were performed with the STATA package 15.0 (2017, Stata Corp. LLC, College Station, TX, USA). Descriptive data were expressed as mean ± standard deviation and 95% confidence interval (CI) or frequency and 95% CI. The comparisons between groups were made by independent *t*-tests or with the Wilcoxon rank-sum test (Mann–Whitney). Chi-square analysis was applied to examine the variations of allele, genotype and genotype frequencies in the different groups. Chi-square analysis was also used to test the Hardy–Weinberg equilibrium for genotypes in all groups of subjects. Odds ratios (ORs) and their 95% CI were computed for VDR alleles using the logistic regression model to estimate association with T2D. A value of *p* < 0.05 was considered statistically significant.

## Results

The participants in this study were men and women with an average age of 66.2 ± 10.0 years. Of the total of 310 subjects, 138 had T2D and 172 were healthy subjects without diabetes.

The subjects with T2D were shown to be more obese than the control subjects, which was represented by higher values for waist circumference, % of altered waist circumference, hip circumference and BMI; they also had higher Homa-IR values than healthy subjects (4.8 ± 9.6 and 1.2 ± 1.1, respectively).

The total average plasma 25(OH)D3 level was 57.5 ± 29.4 nmol/L, and was similar between individuals with T2D and healthy subjects. Overall, 45.6% of the subjects presented vitD levels ≤50 nmol/L and 76.6% presented levels below 75 nmol/L.

The average US-CRP level was 2.9 ± 3.1 mg/L, and was similar between subjects with and without T2D.

Table 1 shows the characteristics of the subjects where the cases and controls are categorized according to the 25(OH)D3 status (vitD ≤ 50 nmol/L and vitD > 50 nmol/L); when comparing the subjects of each group, it can be seen that for both the cases and for the controls in the vitD group under 50 nmol/L the average of this vitamin was significantly lower than in the group of subjects with vitD levels > 50 nmol/L. Within the group of diabetics, those with vitD ≤ 50 nmol/L presented a higher BMI compared with subjects with vitD > 50 nmol/L (32.4 ± 6.6 vs. 30.0 ± 4.3).

**Table 1:** Characteristics of the subjects according to vitamin D levels

	T2D			Controls		
	DVITD ≤ 50	DVITD > 50	*p*-Value	NDVITD ≤ 50	NDVITD > 50	*p*-Value
	*n* = 66	*n* = 72		*n* = 73	*n* = 91	
25(OH)D, nmol/L	32.7 ± 10.0 (30.2–35.2)	74.2 ± 19.1 (69.6–78.8)	<0.001	35.0 ± 11.0 (32.4–37.6)	78.3 ± 22.7 (73.5–83.1)	<0.001
Women, %	68.1 (55.5–79.1)	62.3 (49.8–73.7)	0.460	78.0 (66.9–86.2)	56.0 (45.6–65.9)	0.003
Age, years	61.1 ± 10.7 (58.5–63.8)	62.0 ± 11.9 (59.1–64.8)	0.163	65.7 ± 11.4 (63.0–68.3)	66.7 ± 8.7 (63.2–68.6)	0.239
Waist circumference, cm	100.2 ± 15.0 (96.3–104.1)	97.4 ± 10.8 (94.6–100.2)	0.398	92.0 ± 10.2 (89.3–94.0)	92.1 ± 11.0 (89.7–94.4)	0.959
Hip circumference, cm	107.5 ± 13.7 (104.0–111.1)	103.6 ± 10.9 (100.9–106.4)	0.186	100.9 ± 6.6 (99.4–102.5)	99.4 ± 7.8 (97.7–101.0)	0.088
BMI	32.4 ± 6.6 (30.7–34.2)	30.0 ± 4.3 (28.9–31.1)	0.038	28.4 ± 3.7 (27.5–29.2)	27.7 ± 4.2 (26.8–28.6)	0.105
Fasting insulin, uUI/ml	11.0 ± 10.4 (8.4–13.6)	8.7 ± 8.2 (6.6–10.8)	0.154	5.5 ± 4.8 (4.4–6.7)	4.6 ± 4.3 (3.7–5.5)	0.065
US-CRP, mg/l	3.3 ± 3.0 (2.4–4.1)	3.1 ± 3.2 (2.2–3.9)	0.965	2.3 ± 3.0 (1.6–3.1)	2.7 ± 3.4 (2.0–3.5)	0.923
Fasting glucose, mg/dl	132.6 ± 48.6 (120.7–144.6)	139.6 ± 48.8 (127.7–151.6)	0.262	87.2 ± 13.4 (84.1–90.4)	90.3 ± 15.1 (87.2–93.5)	0.133
Total cholesterol, mg/dl	189.8 ± 46.0 (176.8–202.9)	195.6 ± 42.2 (182.9–208.3)	0.754	194.0 ± 36.4 (184.4–203.6)	195.3 ± 39.0 (186.3–204.3)	0.983
HDL cholesterol, mg/dl	32.6 ± 11.5 (29.3–35.9)	34.2 ± 8.8 (31.6–36.9)	0.266	37.7 ± 10.9 (34.8–40.5)	38.2 ± 11.2 (35.6–40.8)	0.868
LDL cholesterol, mg/dl	128.3 ± 41.4 (116.4–140.3)	126.7 ± 30.5 (117.4–136.0)	0.663	129.3 ± 34.0 (120.3–138.3)	126.3 ± 33.6 (118.5–134.1)	0.630
Triglycerides, mg/dl	139.4 ± 63.4 (121.4–157.4)	152.5 ± 60.4 (134.1–170.9)	0.134	136.1 ± 61.8 (119.8–152.4)	149.4 ± 66.4 (134.4–164.7)	0.232
Homa-IR	3.7 ± 4.0 (2.6–4.6)	3.7 ± 5.0 (2.4–5.0)	0.447	1.2 ± 1.1 (0.97–1.5)	1.0 ± 1.0 (0.85–1.2)	0.143

The data correspond to average ± SD (95% CI)

*p*-value = Wilcoxon rank-sum (Mann–Whitney) test

The FokI polymorphism of VDR was consistent with Hardy–Weinberg equilibrium distribution in cases and controls rs2228570 (*p* > 0.05), whereas the BsmI SNP of VDR rs1544410 was not found in this balance for either cases or controls; the broad genetic mix of the Chilean population may have influenced the Hardy–Weinberg distribution in this case, as similar results have been observed in populations other than the Chilean population^35^.

The frequency of the C allele of the FokI polymorphism was significantly higher in the T2D group than in healthy subjects (*p* = 0.025). The frequencies of the BsmI variant were similar in subjects with and without T2D (*p* = 0.747; Table 2).

**Table 2:** Frequency of genotypes and carriers of FokI alleles and BsmI of VRD by groups with T2D in relation to healthy subjects

		Genotypes			Allele carriers	
Group	*N*	TT *n* (%)	TC *n* (%)	CC *n* (%)	TT *n* (%)	TC + CC *n* (%)
ND	172	53 (30.8%)	81 (47.0%)	38 (22.0%)	53 (30.8%)	119 (69.1%)
T2D	138	28 (20.2%)	86 (62.3%)	24 (17.3%)	28 (20.2%)	110 (79.7%)
DVITD > 50	69	11 (15.9%)	45 (65.2%)	13 (18.8%)	11 (15.9%)	58 (84.0%)
DVITD ≤ 50	66	16 (24.4%)	39 (59.0%)	11 (16.6%)	16 (24.2%)	50 (75.7%)
ND vs T2D			Chi^2^ = 7.38 *p* = 0.025			Chi^2^ = 4.39 *p* = 0.036
ND vs DVITD > 50 vs DVITD ≤ 50			Chi^2^ = 8.51 *p* = 0.074			Chi^2^ = 5.79 *p* = 0.055

		Genotypes			Allele carriers	
Group	*N*	AA *n* (%)	AG *n* (%)	GG *n* (%)	AA *n* (%)	AG + GG *n* (%)
ND	172	97 (56.4%)	49 (28.4%)	26 (15.1%)	97 (56.4%)	75 (43.6%)
T2D	138	77 (55.8%)	36 (26.0%)	25 (18.1%)	77 (55.8%)	61 (44.2%)
DVITD > 50	69	41 (59.4%)	15 (21.7%)	13 (18.8%)	41 (59.4%)	28 (40.5%)
DVITD ≤ 50	66	35 (53.3%)	19 (28.7%)	12 (18.1%)	35 (53.0%)	31 (46.9%)
ND vs T2D			Chi^2^ = 0.58 *p* = 0.747			Chi^2^ = 0.011 *p* = 0.916
ND vs DVITD > 50 vs DVITD ≤ 50			Chi^2^ = 1.70 *p* = 0.790			Chi^2^ = 0.56 *p* = 0.756

*ND* healthy subjects

Consistent with these data, there was an association of the C allele with T2D (OR = 1.74, 95% CI = 1.003–3.084, *p* = 0.036), but not the AG + GG variants for BsmI (OR = 1.02, 95% CI = 0.635–1.649, *p* = 0.916) (Table 3). When analyzing the subjects according to the different genotypes for both VDR polymorphisms, we observed a statistically significant association for subjects with the “TC” genotype and T2D (OR = 2.009, 95% CI = 1.122–3.628, *p* = 0.012). No associations with T2D were observed with the BsmI genotypes (Table 4).

**Table 3:** Odds ratio for carrier genotypes in healthy subjects and T2D

Genotype	ND	T2D	Odds ratio (95% CI)	*p*-Value
FokI				
TT	53 (30.8%)	28 (20.2%)	1	
TC and CC	119 (69.1%)	110 (79.7%)	1.7497 (1.003–3.084)	0.0361
BsmI				
AA	97 (56.4%)	77 (55,8%)	1	
AG and GG	75 (43.6%)	61 (44.2%)	1.0245 (0.6359–1.6494)	0.9160

**Table 4:** Genotypes distribution of the candidate SNP in healthy subjects and T2D

Genotype	ND	T2D	Odds ratio (95% CI)	*p*-Value
FokI (T/C) rs2228570				
TT	53 (31.0%)	28 (20%)	1.00	
TC	81 (47%)	86 (62%)	2.0097 (1.1223–3.6280)	0.0121
CC	38 (22%)	24 (17%)	1.1954 (0.5675–2.5097)	0.6099
BsmI (A/G) rs1544410				
AA	97 (56%)	77 (55%)	1	
AG	49 (28%)	36 (26%)	0.9255 (0.5286– .6138)	0.7722
GG	26 (15%)	25 (18%)	1.2112 (0.6167–2.3735)	0.5476

When performing the logistic regression analysis, we can observe a significant association between carrier of the T > C variant of FokI and T2D, adjusted for vitD, age, obesity (overweight and obesity), seasonality, sex and Homa-IR (Table 5). Here, we show a significant association between the FokI polymorphisms (TC + CC) and T2D with an OR of 1.9001 (95% CI (1.0970–3.6838), *p* = 0.041). No significant associations were observed between the BsmI polymorphism and T2D.

**Table 5:** Logistic regression for T2D and carrier genotypes

T2D	Odds ratio	95% CI	*p*-Value
FokI carriers (TC + CC)	1.9001	1.0970–3.6838	0.041
Vitamin D	0.9870	0.9751–0.9990	0.034
Age	0.9428	0.9163–0.9701	0.000
BMI: overweight	1.1599	0.5057–2.6601	0.726
Obesity	2.6828	1.1707–6.1479	0.020
Season (winter)	0.5649	0.2953–1.0805	0.084
Sex	1.1130	0.6043–2.0498	0.731
Homa-IR ≥ 2.6	5.7738	2.8053–11.883	0.000
BsmI carriers (AG + GG)	1.1972	0.6760–2.1202	0.537
Vitamin D	0.9880	0.9763–0.9999	0.049
Age	0.9448	0.9184–0.9719	0.000
BMI: overweight	1.0916	0.4791–2.4869	0.835
Obesity	2.7069	1.1872–6.1718	0.018
Season (winter)	0.5891	0.3106–1.1171	0.105
Sex	1.1345	0.61715–2.0858	0.684
Homa-IR ≥ 2.6	5.8726	2.8646–12.0391	0.000

FokI carriers: corresponds to the comparison of TC + CC vs TT genotypes

BsmI carriers: corresponds to the comparison of AG + GG vs AA genotypes

BMI: corresponds to the comparison of overweight and obesity vs normal range

Season: corresponds to the comparison of the winter vs summer months

## Discussion

Our study showed that for the VDR-FokI polymorphism, the T allele was dominant (54.3%) in healthy older adults, and the VDR-FokI genotype distribution in this group was 30.8% with TT, 47.0% with TC and 22% with CC. These results are consistent with other research reported by López et al. in 2008, where Chilean adolescents with type 1 diabetes and their association with VDR-FokI were investigated, indicating that the subjects included in the current study represented the group well. In comparison with the controls, the frequency of the FokI allele in subjects with T2D was significantly higher, suggesting an association between the VDR-FokI genotypes “CC and TC” and T2D in older adults of Chilean nationality. The results obtained for VDR-BsmI are also in agreement with a report by García et al.^32^, where three polymorphisms in the VDR gene were studied, focusing on their influence on the immune response in Chilean children with type 1 diabetes. As in the publication by García et al., the data obtained in this investigation do not suggest an association of VDR-BsmI with T2D; this is in contrast with the FokI polymorphism, where the frequencies of the alleles and genotypes for VDR-BsmI in subjects with T2D were not significantly different in comparison with the controls.

Cross-sectional studies have shown that the concentration of 25(OH)D3, a marker that is frequently used to determine vitD status, is lower in individuals with T2D and in individuals with a tolerance to impaired glucose than in those with normal glucose tolerance^36,37^. Prospective studies have shown a significant inverse association between baseline serum 25(OH)D3 levels and the incidence of diabetes^19,38–40^.

Although it was found that the C allele was more frequent in the T2D group compared with the control group, within the group of diabetics, the C allele was no more frequent in individuals with vitD ≤ 50 nmol/L. Therefore, our results suggest that FokI C, especially the TC genotype, is a risk factor for T2D in older adults of Chilean nationality, whereas this association is less clear in diabetic patients with vitD deficiency.

The active form of vitD exerts its effects through the VDR. Several polymorphisms have been described in the VDR gene; among the most studied are FokI (rs2228570), BsmI (rs1544410), ApaI (rs7975232) and TaqI (rs731236). The VDR gene is located on chromosome 12q12-q14 and consists of 14 exons. In the FokI SNP, an alternative ATG start codon is produced in exon 2, the Apa-I, Bsm-I and Taq-I polymorphisms are located near the 3' end of the VDR gene; BsmI and ApaI SNPs are located in intron 8 and TaqI is a silent SNP in exon 9. These SNPs of the VDR gene have been related to chronic diseases of inflammatory, infectious and autoimmune etiology such as prostate cancer, Graves disease, Hashimoto’s thyroiditis, Addison’s disease and type 1 and T2D.

VDR-FokI is the only polymorphism found in the coding sequence of the gene, resulting in different VDR protein products. The FokI SNP consists of a variation of T > C at the start codon of translation in exon 2 within the 5’ end of the gene. The ATG > ACG change results in a protein of 424-amino acids instead of 427; this structure of three amino acids less results in an altered biological activity of the receptor^23,25^. However, the reason for the difference in the activity of the two proteins, for example, whether they bind to 1,25-hydroxyvitamin D differently, is not clearly determined; therefore, the molecular mechanism that marks the association between individuals carrying the T allele of VDR-FokI and T2D has not yet been defined.

There is controversy in the literature about the different impact of FokI SNP in different populations, with some populations not showing association with T2D and other conditions^5,41–43^. This could be attributed to different penetrance, however, a recent meta-analysis found statistical evidence that the C allele of the FokI polymorphism in the VDR gene may be an allele of susceptibility to T2D, especially among Asian populations, but not the BsmI, ApaI and TaqI polymorphisms^44^.

The accumulation of evidence suggests that altered homeostasis of vitD may play a role in the development of T2D and cardiovascular disease^10,36,38,45,46^. Observational studies have reported a consistent association between hypovitaminosis D and the incidence of T2D^47^. Given that patients with T2D have subtle alterations in glucose metabolism long before the onset of the disease, the genetic factors that contribute to its pathogenesis or development could be detected early in the disease process^48^.

Within the limitations of our study, we can mention that the dietary intake of vitD was not registered, however, in Chile, very few foods are fortified with vitD, only some foods such as oils, low-fat milk, butters, the products of national supplementary feeding programs and cereals, the doses only reach 400 IU in the supplementary feeding program for older adults (PACAM). Composed of a mixture of cereals and legumes and a milk drink powder. Both foods are fortified with 25 OH-D and vitamin B12 and are supplied by the Ministry of Health of Chile to people over 70 years enrolled in primary care centers. The PACAM contributes approximately 20% of the daily energy requirements and 50% of the daily requirements of micronutrients, if consumed in the recommended amounts. The contributions of vitD are 4.8 ug/day, which means 32% of the recommendations^49^, in our study only 12% of the participants reported positively consuming PACAM. On the other hand, the Latin American Nutrition and Health Survey (ELNS), conducted on 9000 subjects between 15 and 65 years old in eight Latin American countries (Argentina, Brazil, Chile, Colombia, Costa Rica, Ecuador, Peru and Venezuela) indicates that in Chile the average dietary vitD intake is 2.8 mcg, that is, not exceeding 120 UI daily^50,51^.

The main limitation to this study is the small sample size. Also selection of cases and controls were not population-based and estimates may not be generalizable to the elderly Chilean population. However, its strength lies in being among the first in Latin America, which report this type of association.

A future study in a larger population of T2D with specific groups and detailed information on the levels and activity of VDR, the secretion and sensitivity of insulin and inflammation is necessary in order to understand the mechanisms involved in the association between VDR polymorphisms and diabetes type 2. In conclusion, our study suggests that the C allele (TC/CC) of the VDR-FokI gene is a possible risk factor for T2D in older people living in a community in Santiago de Chile.
